# Impact of “Three Red Lines” Water Policy (2011) on Water Usage Efficiency, Production Technology Heterogeneity, and Determinant of Water Productivity Change in China

**DOI:** 10.3390/ijerph192416459

**Published:** 2022-12-08

**Authors:** Wasi Ul Hassan Shah, Yuting Lu, Gang Hao, Hong Yan, Rizwana Yasmeen

**Affiliations:** 1 School of Management, Zhejiang Shuren University, Hangzhou 310015, China; 2 Department of Management Sciences, City University of Hong Kong, Hong Kong; 3 School of Economics and Management, Panzhihua University, Panzhihua 617000, China

**Keywords:** water usage efficiency, regional heterogeneity, productivity change, DEA

## Abstract

This research evaluates the effects of the Three Red Lines policy on water usage efficiency (WUE), production technology heterogeneity, and water productivity change in 31 Chinese provinces between 2006 and 2020. SMB-DEA, Meta-frontier analysis, and Malmquist–Luenberger index (MLI) techniques were employed for estimation. Results revealed that the mean WUE (2006–2020) in all Chinese provinces was 0.52, with an improvement potential of 48%. Shanghai, Beijing, Shaanxi, and Tianjin were the best performers. The WUE scores before (2006–2011) and after (2012–2020) water policy implementation were 0.58 and 0.48, respectively; on average, there was more than a 9% decline in WUE after the implementation of the water policy. The eastern region has the most advanced water utilization technology as its technology gap ratio (TGR) is nearly 1. The average MLI (2006–2020) score was 1.13, suggesting that the MLI has increased by 12.57% over the study period. Further technology change (TC) is the key predictor of MLI growth, whereas efficiency change (EC) diminished from 2006 to 2020. The mean MLI score for 2006–2011 was 1.16, whereas the MLI Score for the period 2012–2020 was 1.10, indicating a modest decline following the implementation of the water policy. All three Chinese regions experienced MLI growth during 2006–2020, with TC the main change factor.

## 1. Introduction

Water is a transparent fluid that composes the world’s rivers, lakes, oceans, and rainfall. It is an essential item for the living organism. Water scarcity is a huge global challenge in the twenty-first century. The organization of water supplies and water treatment facilities for optimal performance constitutes good water management. Over the past two decades, China’s economy has expanded rapidly, making it the second-largest in the world. This “economic miracle” has lifted hundreds of millions of people out of poverty, but at a heavy price of environmental degradation. The effects of environmental pollution on human health and water scarcity are currently the top concerns of the Chinese government [1]. With the rapid development of the economy and society, water security in China is becoming increasingly severe [2]. Gaps in supply and demand, uneven distribution of water resources, frequent floods, waterlogging disasters, construction of farmland, and water conservancy are still major water-related issues in the country. Water scarcity is a major hurdle in stable agriculture development and national food security. Moreover, China’s economic growth has increased domestic and industrial sewage, which is a major contributor to the depletion of environmental quality [3]. The country’s GDP output per water unit is far below the world average. The average economic productivity of water in the developed world is approximately USD 36 per cubic meter, whereas the economic productivity of water is approximately USD 3.50 per cubic meter in China [4].

Although China has some of the most important rivers in South Asia, the chronological and spatial distribution of water resources is uneven. Among the 31 administrative regions on the mainland, 8 are seriously water deficient, while 20 others face a slight water shortage [5]. These water-scarce provinces face severe challenges for agriculture, industrial, domestic, and even drinking water. These water scarcity challenges ultimately affect people’s health and everyday life and slow the pace of sustainable economic and social goals. In addition, the low level of production technology in agriculture, industry, and domestic usage leads to low water utilization efficiency and wastage of resources [6].

In 2011, the State Council of the CPC Central Committee developed and implemented the “Three Red Lines” strategy and China’s water resources management goals for the next 20 years. The objective was to control the scale of water usage, increase water utilization efficiency, and limit sewage discharge; three “red lines” show the need to regulate water supply, use, and pollution. A further explanation is: (1) controlling the overall quantity of water use, (2) enhancing the efficiency of water usage, and (3) controlling the total amount of pollutant discharge into rivers [7]. By 2030, it was planned to keep China’s total water consumption below 700 billion cubic meters. Water efficiency will be enhanced through water-saving society development. In addition, the effective utilization coefficient of farmland irrigation water would be increased to more than 0.6. Finally, sewage management targets included reducing main pollutant discharges to within the pollutant receiving capacity of the water function zone by 2030 and increasing the water quality standard rate above 95% [8].

**Figure 1:**
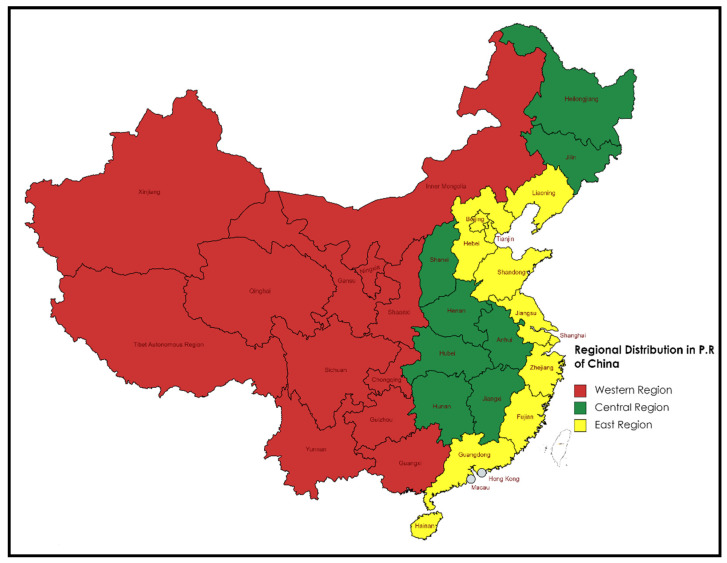
Central, western, and eastern regions in China.

After implementing this policy, Chinese provincial governments have taken serious measures to secure water reservoirs, enhance water usage efficiency, and limit pollutants [7]. However, the degree of this mission’s success has not yet been determined and needs investigation. To this end, our study measured the water usage efficiency through 31 provinces and city-level data from 2006–2020. Therefore, firstly, the study incorporated sewage as a bad output in the estimation process and employed SBM-DEA to gauge the water usage efficiency for extended periods to evaluate the impact of the Chinese government’s water policy. Production technology heterogeneity in different regions of the country could impact water usage efficiency; hence, in the second stage, we used the Meta-frontier method to calculate the technological gap ratio in western, central, and eastern China. This gauges the success rate of different parts of the county in terms of water usage efficiency and provides advice for policy implications for weaker production technology regions to enhance their technology to achieve a higher efficiency frontier. The Malmquist–Luenberger index measures the total factor productivity change in the third stage. It decomposes this productivity change to compute the determinant (technology change or efficiency change) of productivity growth or decline over the study period. It highlights the recommendations for provincial and central governments to either improve their technology or technical efficiency in the conversion process to reduce the inputs (water, labor, and capital) to produce more value-added (GDP) with fewer pollutants (sewage). Finally, to strengthen our findings, the Kruskal–Wallis and Mann–Whitney U tests measure the significant statistical differences in water usage efficiency (WUE), productivity change (MLI), technology change (TC), and technical efficiency change (EC) between the pre- and post-water policy period (2006–2011, 2012–2020) for the three regions of China (see Figure 1). The rest of the study is as follows. Section 2 describes the detailed literature review. Section 3 contains the methodology used in the research. Section 4 provides the inputs–outputs selection and data sources. Section 5 and Section 6 focus on the results and discussion, and conclusion, respectively.

## 2. Literature Review

Data envelopment analysis is a well-known mathematical technique extensively used to gauge the different types of efficiency estimation in various industries and regions globally [9,10,11]. Water usage efficiency is a relatively new phenomenon used to measure the relative efficiency of regions or units that incorporate water consumption with different economic inputs to produce economic value editions [12]. The following literature describes water utilization efficiency in various regions and industries worldwide.

### 2.1. Water Usage Efficiency and Regional Production Technology Heterogeneity

One of the requirements for optimizing water usage efficiency (WUE) is understanding its current state. WUE is also known as economic WUE in social science, defined as the value of the products produced per unit of water usage [13]. According to this conceptual framework, studies have been conducted on water usage efficiency, related policies, and water’s importance in different regions [14,15,16]. It was eventually revealed that using only water as an input would not be capable of generating the required outputs. WUE evaluation also requires other information [17]. As a result, multiple-input model investigations of total factor WUE are becoming more popular. The production environment may differ regionally, which could influence water usage efficiency. In their study, the Meta-frontier technique was used by Rahman et al. (2019) [18] to analyze the effectiveness of 625 farms in Bangladesh that raise phantasies and tilapia in diverse regions.

The results suggest that efficiency varies significantly among various production locations and technologies. According to this research, planners should consider geographical variations in production conditions and the relative performance of different species. Xu et al. [19] studied the agricultural water rebound impact by measuring the causal influence of agricultural water usage efficiency on agricultural water use through panel data from 30 provinces and cities in China between 2000 and 2017. Results revealed a negative correlation between agricultural water usage efficiency and agricultural water usage. However, the average agri-water (water consumption for the agriculture sector) rebound effect is 88.81%. Secondly, there is a regional variation in the rebound effect of increased agricultural water usage efficiency. Veettil et al. [20] used Indian farm-level data to gauge WUE and found that farms operating near the efficiency frontier have higher water needs than those operating at lower efficiency levels. It has been demonstrated that if the pricing system is handled on a volumetric basis, a rise in the price of water will not result in a sizable profit loss, but water consumption would decrease significantly. Similarly, numerous studies employed DEA techniques to estimate the WUE and technological gaps in different regions of the world [21,22,23]. In China, different studies tried to measure the WUE in different regions and industries [24,25,26]. However, a comprehensive study that could analyze the impact of the three red line water safety policies on WUE and regional heterogeneity on provincial data is missing. To this end, this study will evaluate China’s WUE and regional heterogeneity.

### 2.2. Water Usage Total Factor Productivity and Its Determinants

The total factor productivity change could be decomposed into technological and efficiency change, which evaluate the determinant of TFP change. Hu et al. [17] employed DEA to assess the total factor water efficiency for different regions of China. Results of the study revealed that the central region has the lowest water efficiency ranking and accounts for almost 75% of China’s overall water usage. Superior water efficiency, especially in terms of efficient water use, requires adopting more efficient production processes and cutting-edge technology in the central region. Molinos-Senante [27] used data from 1993 to 2016 on the water industry in England and Wales to estimate the total factor productivity. Each year, the water industry witnesses a 6.1% productivity boost, with 1.5% attributable to technological innovations and 4.5% to economies of scale. Assessing the TFP of freshwater efficiency in China from 2004 to 2019, Zhong et al. [28] developed a Meta-frontier Malmquist index (MMI) model that considers regional heterogeneity. The results demonstrated that TFP was highly variable between 2004 and 2012. The total factor productivity (TFP) of inland freshwater was higher than that of coastal freshwater in this study.

Based on the decomposition index, technological progress (TC) and technical efficiency drove freshwater total factor productivity variation. The decomposition index also reveals a lower technical efficiency and a relatively stable managerial efficiency. The coastal areas are near the optimal technical level for freshwater usage, with only a minor gap between the two. On the other hand, inland areas have higher development potential. Wang et al. [29] found that technological advancement has a positive and prominent role in TFP change in China’s freshwater usage efficiency. A study by Molinos-Senante [30] on the water usage TFP change in England shows that productivity increased between 2001 and 2004, with the increase primarily attributable to improvements in efficiency and the decrease attributable to a lack of technical change (except for 2004). Productivity growth slowed between 2005 and 2008, with any efficiency gains erased by the highly negative impact of technological development. The TFP of water usage was measured for Chilean water providers between 2007 and 2018. TFP grew annually by 2.2%, with most of this improvement attributable to greater outputs. The most significant factor in the productivity increase was a shift in scale efficiency, which suggests that water firms may be able to reduce their operating expenses by adjusting the size of their operations [31]. Oulmane et al. [32] demonstrated that increasing financial support for WST increases water’s total factor productivity. Adopting these methods enables reduced water consumption during crop production. This research indicates that WST may affect productivity increases in total factor productivity. Further, numerous studies gauge the impact of technology and technical efficiency on water and environmental TFP growth [33,34,35,36,37]. Our study investigates the impact of the Chinese government’s three red line water security policy on water usage TFP and explores its determinant (technology or efficiency).

## 3. Methodology

There are numerous techniques available to measure the efficiency of homogeneous decision-making units. Data envelopment analysis (DEA) and stochastic frontier analysis (SFA) are extensively used in the literature. DEA has an advantage over SFA because it does not require functional form specification and can gauge efficiency through linear programming. This study employed advanced DEA models, the super-efficiency SBM model with undesirable outputs, the DEA Meta-frontier model, and the Malmquist–Luenberger index. Further, when the data are non-normally distributed, Kruskal–Wallis and Mann–Whitney tests are more appropriate to measure the statistical difference between the two populations. The detailed properties and advantages of the used techniques are given below.

### 3.1. Super-Efficiency SBM Model with Undesirable Outputs

The super-efficiency SBM model, proposed by Tone [38], is a non-radial DEA model that allows for simultaneous consideration of input and output to evaluate the efficiency of homogenous decision-making units (DMUs). The super-efficiency SBM model overcomes the shortcoming of the radial DEA model, which lacks slack variables in the estimation process. Super-SBM can overcome the shortcomings of radial measurement and efficient DMUs can be distinguished from one another. Tone [39] developed the Undesirable Super-SBM model. The proposed model is a pioneer in the study to account for bad output and rank efficient units. Details of the model are as follows:

If there are n DMUs and each one has m inputs, then *s*_1_ and *s*_2_ are, respectively, the good and bad outputs. The input–output matrix has the formulas$\;X=\left[x_{1}\ldots x_{n}\right]\in R^{m\times n},Y^{nd}=\left[y_{1}^{d}\ldots y_{n}^{d}\right]\in R^{s_{1}\times n}$, and $Y^{u}=$ $\left[y_{1}^{A}\ldots y_{n}^{ut}\right]\in R^{s_{2}\times n}$. The expression of the super-efficiency SBM model with bad output is shown below.

(1) $$\rho^{*}=\frac{\frac{1}{m}\sum_{i=1}^{m}\left(\frac{\bar{x}}{x_{ik}}\right)}{\frac{1}{\left(s_{1}+s_{2}\right)}\left(\sum_{r=1}^{s_{1}}\frac{\bar{y^{d}}}{y_{rk}^{d}}+\sum_{t=1}^{s_{2}}\frac{\bar{y^{u}}}{y_{rk}^{u}}\right)}\;s.t.\;\left\{ \begin{array}{l} \bar{x}\geq \overset{n}{\underset{j=1,4k}{\sum }}x_{ij}\lambda_{j};i=1,2,\ldots m\\ \bar{y^{d}}\leq \overset{n}{\underset{j=1,\neq k}{\sum }}y_{rj}^{d}\lambda_{j};r=1,\ldots ,s_{1}\\ \bar{y^{u\mu }}\geq \overset{n}{\underset{j=1,\neq k}{\sum }}y_{tj}^{u}\lambda_{j};t=1,\ldots ,s_{2}\\ \lambda_{j}\geq 0,j=1,2,\ldots n,j\neq 0\\ \bar{x}\geq x_{ik};y^{d}\leq y_{rk}^{d};\bar{y^{\mu }}\geq y_{dk}^{u} \end{array}\right.$$

The slack variables of input, desirable output, and undesirable output, respectively, are $\overset{¯}{x},\bar{y^{d},}$ and ${\overset{¯}{y}}^{u\;}$in the formula; λj is the weight vector; and $\rho^{*}$ is the model’s optimal solution, with the DMU effective when $\rho^{*}\geq 1$.

### 3.2. DEA Meta-Frontier Model

The Meta-frontier model allows for more precise estimations of DMU efficiency evaluations with different groups. Comparing DMUs within the same group is preferable to ensure that they all have access to the same level of technology. To determine how far the various groups are in terms of technological development, the technology gap ratio (TGR) can be used. TGR can be presented for a specific group [40,41].

(2) $$TGR=\frac{MWUE}{GWUEi}$$

The evaluation considers the WUE of all DMUs, where GWUE i represents the water usage efficiency of DMUs within a particular group category and MWUE represents the Meta-WUE of DMUs within a particular technical level. The TGR employs a distance metric to determine how close a Meta-frontier technology is to a group’s frontier technology [42]. TGR is commonly used to evaluate regional disparities. Further, TGR =1 shows no technological gap between a group and the Meta frontier.

### 3.3. Malmquist–Luenberger Index

Chung et al. [43] renamed the Malmquist index as the Malmquist–Luenberger index because it included an undesirable directional distance function. The new metric is decomposed into two factors: technological change (TC) and efficiency change (EC). The ML index between *t* and *t* + 1 is represented as:

(3) $$ML^{t+1}={\left\{\frac{\left[1+\vec{D_{0}^{t}}\left(x^{t},y^{t},b^{*};y^{t},-b^{t}\right)\right]}{\left[1+\vec{D_{0}^{t}}\left(x^{t+1},y^{+1},b^{t+1};y^{t+1},-b^{t+1}\right)\right]}\times \frac{\left[1+\vec{D_{0}^{t+1}}\left(x^{t},y^{t},b^{t};y^{t},-b^{t}\right)\right]}{\left[1+\vec{D_{0}^{t+1}}\left(x^{t+1},y^{+1},b^{++1};y^{t+1},-b^{s+1}\right)\right]}\right\}}^{1/2}\;$$

(4) $$EC^{,x+1}=\frac{1+\vec{D_{0}^{t}}\left(x^{t},y^{t},b^{\prime };y^{t},-b^{2}\right)}{1+\vec{D_{0}^{t+1}}\left(x^{t+1},y^{t+1},b^{+1};y^{k+1},-b^{t+1}\right)}\;$$

(5) $$TC^{4+1}={\left\{\frac{\left[1+\vec{D_{0}^{t+1}}\left(x^{t},y^{t},b^{t},y^{t},-b^{t}\right)\right]}{\left[1+\vec{D_{0}^{t}}\left(x^{t},y^{\prime },b^{t};y^{t},-b^{\prime }\right)\right]}\times \frac{\left[1+\vec{D_{0}^{t+1}}\left(x^{t+1},y^{++1},b^{'+1};y^{t+1},-b^{t+1}\right)\right]}{\left[1+\vec{D_{0}^{'}}\left(x^{t+1},y^{t+1},b^{t+1};y^{t+1},-b^{++1}\right)\right]}\right\}}^{\frac{1}{2}}\;\;$$

where *x*, *y*, and *b* represent input, desirable output, and undesirable output, respectively; (D 0t) $\vec{D_{0}^{t}}\left(x^{2},y^{r},b^{r};y^{t},-b^{t}\right)$ and $\vec{D_{0}^{t+1}}(x^{s+1},y^{t+1},b^{t+1};y^{t+1},-$ $b^{t+1}$ ) are the distance functions of periods *t* and *t* + 1, respectively; $\vec{D_{0}^{t}}$ $\left(x^{s+1},y^{s+1},b^{t+1};y^{s+1},-b^{t+1}\right)$ is the distance function of the *t* + 1 period under the technical condition of the *t* + 1 period; and $\vec{D_{0}^{t+1}}\left(x^{t},y^{t},b^{t};y^{t},-b^{t}\right)\;$is the distance function of the t period under the technical condition of the *t* + 1 period. Total factor energy efficiency (ML > 1, ML = 1, and ML < 1), technical efficiency (EC > 1, EC = 1, and EC < 1), and technological development (TC > 1, TC = 1, and TC < 1) all show growth, stability, and decline, respectively.

### 3.4. Mann–Whitney U and Kruskal–Wallis Tests

Wilcoxon’s Mann–Whitney U test [44,45] is a non-parametric test comparing the results of two independent groups. The Mann–Whitney U test, also known as the Mann–Whitney Wilcoxon Test or the Wilcoxon Rank Sum Test, determines whether two samples come from the same population. In this test, the medians of the two populations are compared. However, if there are more than two independent groups, the Kruskal–Wallis test [46] is used to measure the statistically significant difference. We used the Mann–Whitney U test to measure the statistically significant difference among the mean water usage efficiency (WUE), MLI, TC, and TE between two periods, 2006–2011 and 2012–2020. Therefore, our null hypothesis for the Mann–Whitney U test is as follows:

**H1:**  *The distribution of average WUE is identical for both time periods (2006–2011 and 2012–2020).*

**H2:**  *The distribution of average MLI is identical for both time periods (2006–2011 and 2012–2020).*

**H3:**  *The distribution of average TC is identical for both time periods (2006–2011 and 2012–2020).*

**H4:**  *The distribution of average EC is identical for both time periods (2006–2011 and 2012–2020).*

The Kruskal–Wallis test was used to determine whether the mean water usage efficiency (WUE), MLI, TC, and TE across all three regions differ significantly. The following are our null hypotheses for the Kruskal–Wallis test:

**H5:**  *The distribution of average WUE is identical across China’s three distinct regions.*

**H6:**  *The distribution of average MLI is identical across China’s three distinct regions.*

**H7:**  *The distribution of average TC is identical across China’s three distinct regions.*

**H8:**  *The distribution of average EC is identical across China’s three distinct regions.*

## 4. Inputs-Outputs Selection and Data Sources

The selection of correct inputs–outputs data for efficiency and productivity change is essential for accurate DEA estimation results [47]. Water efficiency cannot be measured with only single water input. Therefore, labor and capital unanimously agreed on inputs with water consumption to produce economic value edition GDP [17]. However, not incorporating undesirable input in the estimation process could generate biased results in DEA evaluation [48]. To this end, the inputs and outputs given in Table 1 are selected from previous studies. Data for 31 Chinese provinces and administrative units were taken from China’s statistical books for 2006–2020. The unit of labor is a person aged between 15–64, capital stock is CNY 10,000, water consumption is 100 million cu. m, and unit of GDP is CNY 100 million. Finally, the unit of sewage is 10,000 tons. Sewage water includes solid waste and pollutants and the unit is 10,000 tons.

## 5. Results and Discussion

Section 5 presents the WUE, production technology gaps, and productivity change results of the study.

### 5.1. SBM-DEA Results

**Figure 2:**
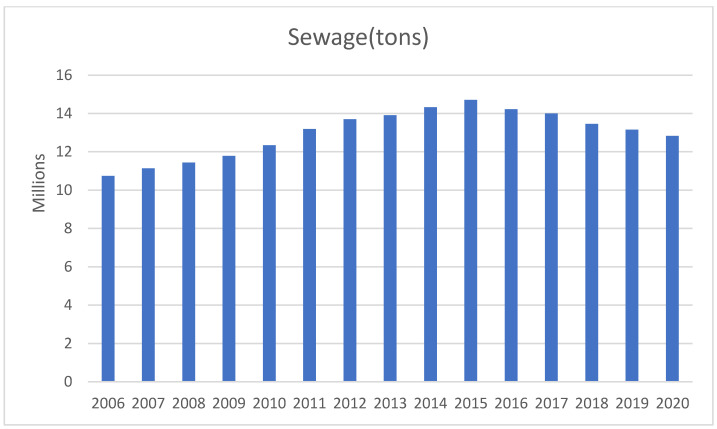
Total sewage in 31 provinces of China.

Since the Chinese government issued and implemented the “Three Red Lines” water policy in 2011, provincial governments have actively responded to the central government’s call. In addition, efforts have been made to promote the remediation measures of water supply, water use, and water pollution; however, the policy’s implementation effect does not appear optimal. Figure 2 shows the sewage discharge of 31 provinces in China from 2006 to 2020. From 2011 to 2015, the sewage discharge increased and reached its peak in 2015. The policy does not appear to reflect its enforceability. After 2015, sewage discharge began to reduce, but the pace was gradual and slow. The policy demonstrates that the delay in sewage treatment is severe and insufficient. Therefore, to gauge the impact of bad output (sewage) on water usage efficiency, we employed the Super-SBM DEA model with undesirable output.

**Figure 3:**
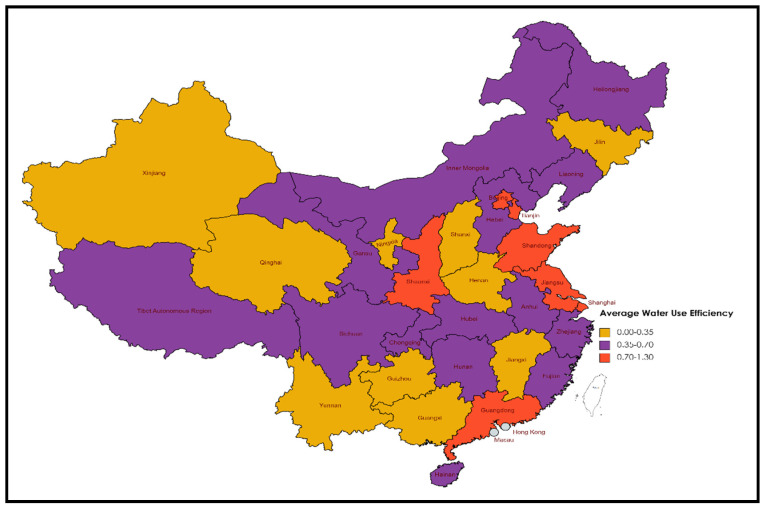
Average water usage efficiency level (2006–2020) grouped by province.

**Figure A1:**
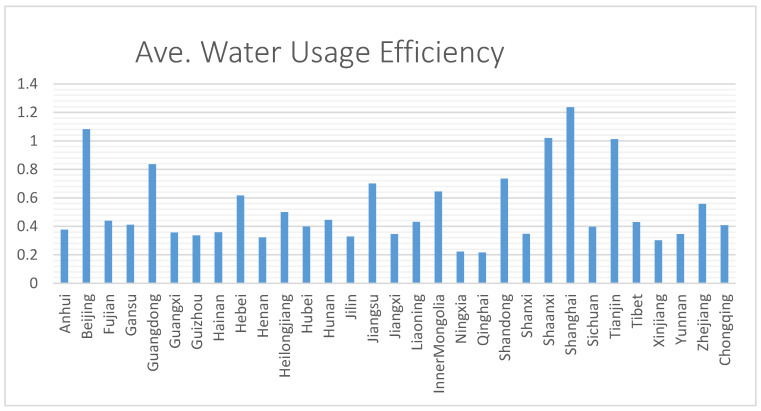
Average WUE across 31 Chinese provinces (2006–2020).

The model used economic inputs with water consumption to produce GDP and sewage. Figure 3 and Figure A1 show the water usage efficiency levels of 31 provinces in China from 2006 to 2020. The mean WUE in all Chinese provinces is 0.52, with an improvement potential of 48%. The results further show that, compared with other provinces in China, Shanghai has the highest average WUE value of 1.236. In addition, there are three provinces with an average WUE greater than 1, namely, Beijing (1.08), Shaanxi (1.02), and Tianjin (1.01). The results demonstrate that these provinces or municipalities can adopt sustainable growth policies and increase water efficiency throughout the study period. Shanghai’s economy is relatively developed, and its water resources utilization technology and management system are advanced. In addition, Beijing, Shaanxi, and Tianjin have always had serious water shortages. The government has strict control over water resources, complete management systems, and a higher water utilization ratio than other provinces. The results show that most provinces in central and western China are inefficient, and their average WUE score is less than 0.5, which indicates that the energy efficiency level of these provinces or cities still has a 50% improvement potential. Inefficient provinces and cities could reduce their water and economic inputs to achieve an efficient frontier demonstrated by efficient DMUs. Alternatively, they could retain the inputs and improve their economic output with the least sewage to become efficient. The efficient province sets the benchmark policies and practices for inefficient provinces to follow and improve their WUE. Deng et al. [26] had similar conclusions and advised similar policy implications for inefficient provinces. However, the WUE is diverse for different periods and provinces, as a change in WUE fluctuates over time and geographical regions.

To check the impact of water policy on water usage efficiency over time, we also compared the annual average WUE from 2006 to 2020 (See Table 2). The results demonstrate that before 2011, China’s annual average WUE showed a fluctuating downward trend. After the announcement and implementation of the “three red lines” water policy in 2011, the annual average WUE showed an upward trend from 2012 to 2015. However, afterwards, a gradual and continued decline in WUE was witnessed. The average WUE before the water policy (2006–2011) was 0.58, indicating a 42% potential efficiency improvement in the water utilization process. The mean efficiency score after the water policy implementation was 0.48. It demonstrates that, on average, there was more than a 9% decline in water usage efficiency after implementing the water policy. It further increases the potential for improvement in WUE to 52%. These results indicate that although there is a decline in sewage with time, WUE declined over the study period, and the mission to increase water efficiency was not achieved compared to its targets. In addition, Chinese provinces could not use water resources efficiently with sustainable development. Scarce water resources, lack of advanced sewage treatment technologies, excessive labor, and capital inputs with region gaps to optimum value addition (GDP) are the potential causes of the decline in water usage efficiency. Byrnes et al. [49] concluded that water scarcity, technology, and excessive inputs are the main hurdles to improving water usage efficiency. Sewage treatment technologies, efficient water usage in agriculture through leveling fields, surge flooding, capture and reuse of runoff, drip irrigation, spray irrigation technologies, and domestic and industrial water safety through public awareness campaigns are possible solutions to increase water usage efficiency in China. Further, reducing the economic inputs (capital and labor) through labor skill programs and efficient utilization of public funds could increase the WUE.

### 5.2. Meta-Frontier DEA Results

Production technology heterogeneity impacts the resource utilization capability of any region or firm, ultimately influencing the production process’s efficiency level. Thus, without distinguishing the technology gap ratio in different regions of China, our WUE results could be biased. Therefore, we employed Meta-frontier analysis to accurately evaluate water resources utilization efficiency and elaborate on the technology heterogeneity in different Chinese regions. Production technology regional heterogeneity in three different regions (see Figure 1) of China was estimated to advise the policy implication for the Chinese government to reduce the technology gap ratio in different regions of the country. The MWE, GWE, and TGR associated with the group frontier, Meta frontier, and technology gap ratio are shown in Table 3. To explain the results in Table 3, we take the example of Anhui Province, located in the central region. Results revealed that the average water usage efficiency (GWE) under the group frontier was 0.91. It shows that compared to other provinces in the central region, the WUE of Anhui was high, with only a potential of 9.2% to reach the efficient frontier in its central group. The core region’s technology serves as a benchmark for Anhui. According to the Meta-frontier analysis, the average MWE for water resources in Anhui was 0.38, with a growth potential of 62%; this is much greater than the group frontier. Compared to other provinces, Anhui has a greater group frontier efficiency but a poor WUE in the Meta frontier. There was also a significant difference in water-use efficiency between the central region and the rest of the country, as indicated by the technological gap ratio (TGR) of 0.43, which is significantly less than 1. The situation in most provinces of China is similar to that in Anhui. The Meta-frontier efficiency in water resources is lower than that of the group frontier. Therefore, Meta-frontier results conclude that the national technology level is the highest, while the group frontier only indicates the region’s best technology. Eastern provinces, such as Beijing, Shanghai, Shaanxi, and Tianjin, have demonstrated higher efficiency within the group and nationally. It shows that these provinces or cities are the benchmark and demonstrate best practices and policies for the rest of the provinces that cannot achieve effect frontier. Moreover, Table A2 and Table A3 show the results of all three regions for the Meta frontier and group frontier over the study period. The findings show that the eastern region recorded the highest water usage efficiency in the Meta frontier with a value of 0.74. It demonstrates that eastern provinces are more efficient in water usage than central and western provinces. The WUE values of the central and western provinces were 0.38 and 0.42, respectively, showing that the central and western regions are less efficient than eastern coastal regions. As eastern regions obtained more advanced water treatment technologies, more sophisticated water and economic practices and policies are the main causes of higher WUE values in the region. The regional difference in water usage efficiency was also measured by numerous studies and concluded similar results and suggestions to improve it [25,50,51]. Comparing the results of the group frontier, the central region recorded a higher WUE = 0.879, which shows that central provinces perform better in their group and only need 12.1% potential to achieve an efficient frontier. The average WUE of the western region was 0.84, while the eastern region recorded a 0.74 score. Meta frontier and group frontier results explain that although central and western provinces secured lower scores in the Meta frontier, their own groups’ average WUE was higher. These results discloses two facts: Firstly, gaps between the efficiency scores in the eastern region are very high, making the average value lower in groups. For example, the WUE of Beijing and Tianjin was 1.08 and 1.02, respectively; in contrast, the WUE scores of other eastern provinces such as Fujian, Hainan, and Liaoning were 0.44, 0.36, and 0.43, respectively. This indicates an enormous efficiency gap, which is why group efficiency declines. It also sheds light on the fact that not all the eastern provinces display higher WUE scores. Secondly, the WUE in central and western regions almost perform equally as there are not huge efficiency gaps between the provinces, which increases their group frontier efficiency. Our conclusion is backed by Chen et al. [52], who discuss the possible reasons for efficiency heterogeneity in different regions and its impact on the efficiency level in the group and Meta frontier.

**Figure 4:**
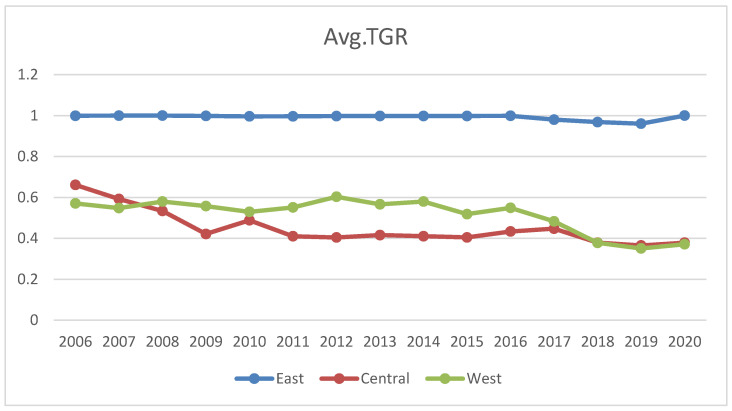
Technology gap ratio between three different Chinese regions (2006–2020).

The technology gap ratio (TGR) is an indicator explaining the production technology gaps between different regions. Figure 4 shows the average TGR of China’s eastern, central, and western regions from 2006 to 2020. The TGR of water resources utilization in the eastern region is far greater than that in the western and central regions; during the entire sample period, the technology gap ratio in the east was maintained near 1. The eastern provinces, therefore, possess the most advanced water resources usage technology. As the initiator and earliest adopter of modern water use technology, the east holds China’s most advanced water resources development and management system. The fact that eastern provinces perform well under the Meta frontier outlined above supports this point. The technology gap ratio in the west was 0.52, which lagged behind that in the east but was superior to that of the central region (0.45). Another bunch of studies endorsed our findings of technology gaps in different regions of China [53,54,55]. It is suggested that the central and western regions develop through R&D or acquire advanced water conversion technologies from eastern regions to narrow down their TGR. Otherwise, the water resources in the central and western regions will face more scarcity, and sewage will be discharged into the water reservoirs, contaminating the underground water resources and ultimately affecting humans and other living species. Technological development can make water usage more efficient in domestic, industrial, and agricultural sectors. Similar recommendations were made to improve the WUE through modern technological development in different studies globally [56,57,58].

### 5.3. Malmquist–Luenberger Index Results

**Figure 5:**
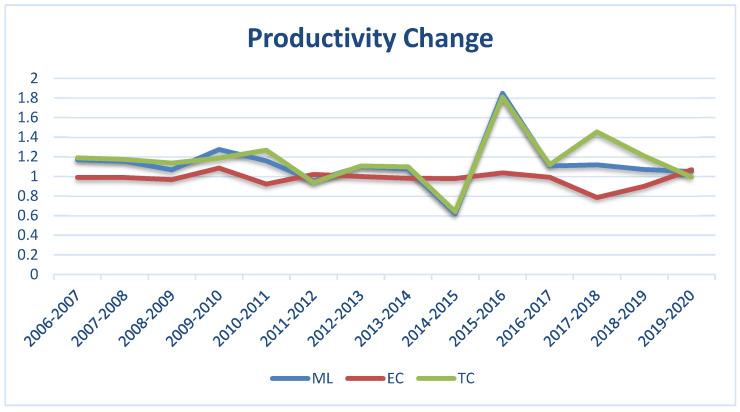
Average Malmquist–Luenberger index change, efficiency change, and technological change in Chinese provinces (2006–2020).

We analyzed the total factor water productivity changes in various cities, provinces, and regions using the Malmquist–Luenberger index. Figure 5 indicates that the average value of the MLI (2006–2020) was 1.13, representing an increase of 12.57% over the study period. Further decomposing the MLI into TC and EC reveals that the MLI changes were mainly determined by technological change. The average value of technical change was 1.1651, which indicates that technological progress has improved by 16.50%; however, the average value of efficiency change EC was 0.98, which indicates that efficiency change has decreased by 2.12%. It further demonstrates the MLI, EC, and TC trends during the study period. These results show that although China’s water productivity improved during this period, the main determinant is TC, as EC is below 1, indicating a decline. Therefore, on the national level, the Chinese government needs to improve the technical efficiency in water conversion to further enhance productivity growth. Efficient operational and management practices and inputs (water, labor, capital) reduction and eliminating undesirable outputs could help achieve the desired targets. The importance of EC in the growth of MLI and recommendations and suggestions to increase efficiency change in different regions was further discussed in many research studies [59,60,61].

Further, to explain the water policy on water productivity change, we divide the results of MLI into two time chunks. The MLI score for 2006–2011 was 1.16, showing 16.4% growth, while the 2012–2020 MLI score was 1.1045, showing 10.45% growth. These results revealed that MLI declined after water policy implementation. The main culprit in this decline is EC, which decreased from 0.99 to 0.97 in two periods. Inefficient management practices, a wastage of input resources, and a decline in output are the potential causes of efficiency decline. Similar kinds of reasons to improve the efficiency change in water utilization of different sectors of an economy were discussed in different studies [62,63].

We compared the regional MLI of eastern, central, and western China from 2006 to 2020. An evaluation of water resources utilization efficiency and the technological level in various regions of China would assist in establishing policies for water resources usage and pollutant treatment. Table 4 shows the mean MLI of the three regions in China over the study period. The western region was the best-performing region, with an average MLI score of 1.15. The eastern and central regions had ML scores of 1.12 and 1.10, respectively. In addition, the three regions all had adequate technological growth rates. The western region had the highest technological growth of 1.19, followed by the eastern region at 1.16 and, finally, the central region at 1.13. These results clearly show that during the study period, the average growth rate of technology in the western provinces of China was 19.35%, taking the lead. The eastern and central regions also showed technological growth, with increases of 16% and 13%, respectively. The average efficiency change (EC) score of the eastern, western, and central regions (0.98, 0.98, and 0.97) was below 1, clearly explaining the decline in EC over the study period. Therefore, we conclude that the growth in MLI is mainly due to TC in all three regions with different technology levels, while EC declined in all three regions. Therefore, to further enhance MLI growth, all three regions need to increase their EC by increasing efficiency through best management practices and operational strategies in the water conversion process. Our results are backed by studies that elaborate on the importance of EC in MLI growth [64,65]. Beijing, Jiangsu, Shanghai, Shaanxi, and Chongqing are the only five provinces in all three regions whose EC is greater than 1. It indicates that these administrative units grew in technical efficiency; therefore, their policies and operational strategies are the benchmarks for all other provinces if they want to improve their EC. Mainly due to technological progress, the output of water resources utilization has improved. In contrast, water resources productivity has declined due to the overall decline in technical efficiency. Therefore, we suggest all three regions improve water productivity through technological innovation and management efficiency. The central region needs to formulate and implement policies to reduce regional differences. Different studies had similar results, which stress narrowing down the regional heterogeneity to increase the MLI [66,67,68].

### 5.4. Mann–Whitney U and Kruskal–Wallis Test Results

**Figure 6:**
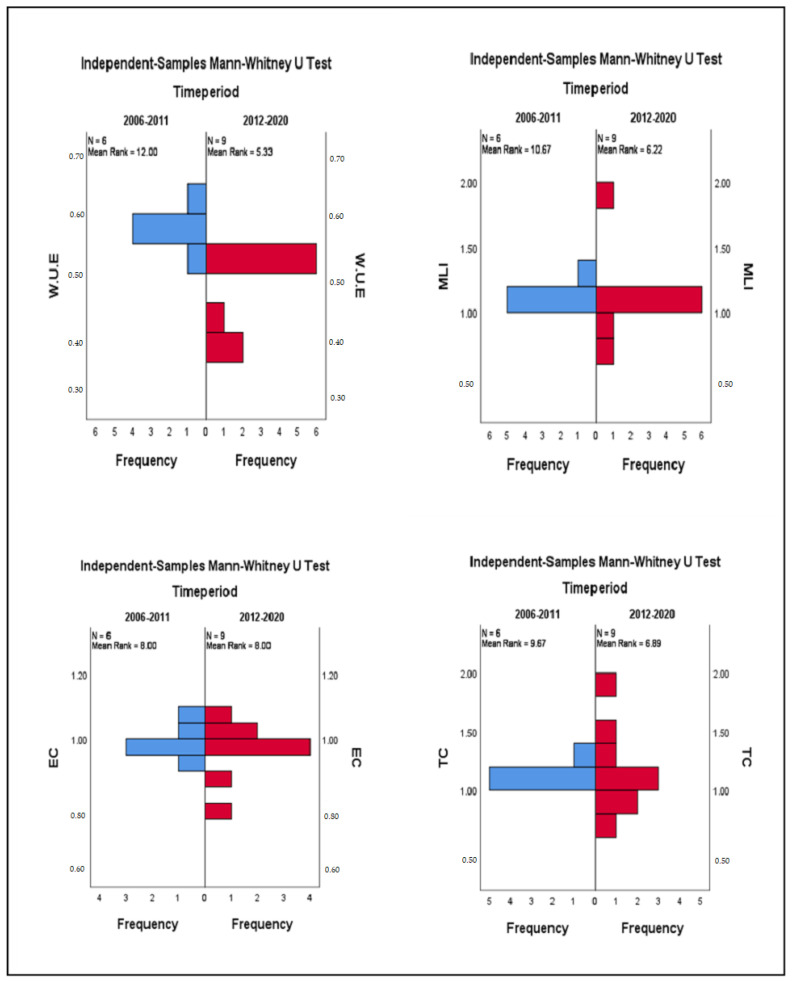
Distribution of average WUE, MLI, EC, and TC across different periods.

To further strengthen the results, the Mann–Whitney U test of independent samples was applied to find the significant statistical difference between the average WUE of two time periods (2006–2011 and 2012–2020). The results of Table 5 and Figure 6 show that the significance value of 0.003 is less than 0.050; therefore, we reject the first null hypothesis, which states that the distribution of the average WUE is identical for both periods (2006–2011 and 2012–2020). It proves that the mean WUE declines after water policy implementation and significantly differs from the mean WUE value before the policy announcement. Further studies conclude that water usage efficiency in China decreased over the study period [69,70].

To assess if a statistically significant difference exists between the average MLI before and after the 2011 water policy, we divided the time into two chunks and applied the second hypothesis. The distribution of Avg. MLI results show that the significance value of 0.066 is greater than 0.050; therefore, we retain our second hypothesis and conclude that although there was a gradual decline after 2011, no significant difference was found between the MLI of the two periods (2006–2011 and 2012–2020). It shows that although there was a deterioration in MLI, on average, no significant difference was found over the study period. A similar conclusion was argued in recent studies about the MLI change in Chinese water usage efficiency [71,72].

There are two determinants for the change in MLI (EC and TC). Therefore, we have developed third and fourth hypotheses to test the significant difference between technology change and efficiency change in the two time periods. The results in Table 5 and Figure 6 show no significant difference between the levels of technological change in the two time periods. Further, efficiency has not changed significantly during this period, as the significance values are greater than 0.50, so we retain the third and fourth null hypothesis and conclude that the mean EC and TC for the two periods does not show any statistically significant difference. Slow technological and efficiency change was also found in recent studies evaluating water utilization in different sectors of the Chinese economy [73,74].

**Figure 7:**
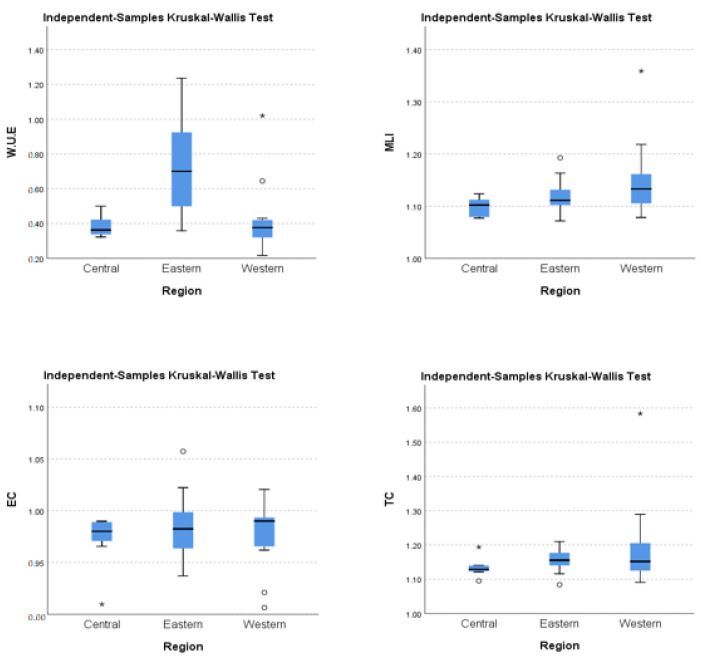
Distribution of average WUE, MLI, EC, and TC across the three regions of China.

Results for the three different Chinese regions demonstrate that WUE, MLI, TC, and EC of the east, central, and west are diverse and at different levels, as water usage efficiency, technology, and MLI is higher in the eastern region. Therefore, to strengthen our results’ validity and for robustness analysis, we applied the Kruskal–Wallis test to gauge the statistically significant differences among WUE, MLI, TC, and EC in all three regions of China. Table 6 and Figure 7 show the results of the Kruskal–Wallis test. As the significance value 0.02 is less than 0.050, we reject our fifth null hypothesis that the distribution of Avg. WUE is the same for three different regions of China. Furthermore, we conclude that water usage efficiency is significantly different among the three regions of China. Similarly, the significance values of the sixth and seventh hypotheses are below the significance level of 0.050. Hence, we reject our null hypothesis stating that the distribution of the Avg. MLI change and TC are the same for three different regions of China. This proves that water productivity change and technology change are significantly different for the three regions of China. Average WUE, MLI, and TC are different in the three regions of China and were also proved by researchers in different periods [75,76]. However, our last hypothesis, that the distribution of the Avg. Efficiency change is the same for the three regions of China, was not rejected as the significance value is greater than 0.050. Hence, we conclude that EC in all three regions is not significantly different.

If we describe the WUE results of each region in detail, the eastern region is much higher than the central and western regions. The main reason for the low efficiency of the central and western regions is the excessive usage of inputs, including water resources, capital, and labor. A further discharge of unprocessed sewage and technological gaps are also potential causes. Sun et al. [77] also devised similar recommendations to improve WUE and technological gaps in different regions of China. To improve the MLI, TC and EC are the main determinants. The western and central regions’ MLI can be enhanced by upgrading their technological infrastructure and improving the technical efficiency in the conversion processes.

## 6. Conclusions and Recommendations

This article examined the impact of the Chinese government’s Three Red Lines water policy on water usage efficiency, technology heterogeneity, and the productivity of water resources utilization in China, taking sewage discharge into account. Total water consumption, labor, and capital were chosen as input indices, and regional GDP and sewage emissions were selected as output indices. Firstly, the WUE values of all provinces were estimated using the Super-SBM-DEA model to determine efficiency changes across the study period (2006–2020) and among the three regions. The regional variation in production technology of the three Chinese regions was measured through Meta-frontier analysis. The ML productivity index and its components (TE and EC) were gauged using MLI to measure each province’s productivity and technical efficiency changes. Finally, Kruskal–Wallis and Mann–Whitney U tests were used to gauge the statistical difference in mean WUE, MLI, TE, and EC among pre- (2006–2011) and post (2012–2020)-water policy and three different regions. The main conclusions of this study are as follows:

(1) Research shows that the mean WUE (2006–2020) in all Chinese provinces was 0.52, with an improvement potential of 48%. Compared with other provinces in China, Shanghai had the highest average WUE value of 1.2. In addition, there were three provinces with an average WUE greater than 1, namely, Beijing (1.08), Shaanxi (1.02), and Tianjin (1.01). After the announcement and implementation of the “Three Red Lines” water policy in 2011, the annual average WUE showed an upward trend from 2012 to 2015. However, afterwards, a gradual and continued decline in WUE was witnessed. The average WUE before the water policy (2006–2011) was 0.5787. The mean efficiency score after the water policy implementation was 0.48. It demonstrates that, on average, there was more than a 9% decline in water usage efficiency level after the implementation of the water policy. Results revealed that the mission to increase water efficiency was not achieved in relation to its targets. In addition, Chinese provinces could not use water resources efficiently with sustainable development. (2) Meta-frontier analysis proved that there are giant production technology gaps in the three regions of China. The eastern region recorded the highest water usage efficiency in the Meta frontier with a value of 0.74. It demonstrates that the eastern region is more efficient in water usage compared to the central (0.38) and western (0.42) regions. Eastern provinces, such as Beijing, Shanghai, Shaanxi, and Tianjin, have demonstrated higher efficiency within the group and national level; therefore, they are announced to be the benchmark for inefficient provinces. The TGR in the eastern region was far greater than that in the western and central regions; during the entire sample period, the technology gap ratio in the east was maintained near 1. This proved that the eastern provinces have the most advanced water resources utilization technology. The technology gap ratio in the west was 0.5158, which lagged behind that in the east but was superior to the central region (0.45). (3) The average MLI (2006–2020) was 1.12, indicating that MLI has increased by 12.57% over the study period. Further decomposing the MLI into TC and EC revealed that the MLI changes were mainly determined by technological change. The average value of technical change was 1.1651, which indicates that technological progress has improved by 16.50%; however, the average value of efficiency change (EC) was 0.98, indicating that the efficiency change has declined by 2.12% over the period. The MLI score for 2006–2011 was 1.16, while the MLI Score for the period (2012–2020) was 1.10, showing a small decline after water policy implementation. EC was the main culprit in MLI’s decline over the period. The western region was the best-performing region, with an average MLI score of 1.1498. The remaining eastern and central regions had ML scores of 1.12 and 1.10, respectively. TC was the main determinant in the growth of all three regions. (4) A statistically significant difference in mean WUE was found among pre- and post-water policy announcement and implantation. For MLI, TC, and EC, no significant difference was found for both periods. In contrast, the mean WUE, MLI, and TC among the three different regions are significantly different, while the mean EC is the same across three different regions.

Based on these findings, the following policy recommendations can be made. At present, China’s agricultural production is a major user of water resources. Therefore, fostering innovation in agricultural production methods and technology, on the one hand, and raising public knowledge about the importance of water conservation could increase water usage efficiency. In particular, the following actions are recommended:

(i) Developing regionally unique water use and water conservation strategies. We cannot analyze GDP growth in isolation while neglecting the negative consequences of bad output on production. Therefore, the industrial structure must be reformed, firms with high pollution and low value-added manufacturing must be punished, and businesses with low water consumption and high value-added production must be encouraged for further investments. (ii) Enhancing investment in education, science, and technology. Specifically, new water conservation courses should be established to increase citizens’ water conservation awareness. Increase science and technology contributions to modernize technologies, improve water conservation, and use efficiencies. (iii) The Chinese government should strictly enforce sewage treatment policies on industrial units to reduce polluted water discharge. The western and central regions should develop or acquire production technologies to narrow their TGR. Modern irrigation technologies should be developed and applied to secure excessive water utilization in agriculture. (iv) Water resources productivity could be further enhanced through EC development. Therefore, provincial governments need to improve their operational strategies and increase technical efficiency to reduce inputs such as water consumption, labor cost, and capital with sustainable economic growth and least emissions. Sewage data for the agriculture sector are missing, which is a study limitation. In the future, if sewage data from the agriculture sector is available, the water usage efficiency and productivity change of the agriculture sector can be gauged.

## Figures and Tables

**Table 1:** Inputs and Outputs used for WUE and productivity change estimation.

Inputs	Outputs
Labor: Population (persons) aged 15–64	Expected output Real GDP (CNY 100 million)
Capital: Capital stock (CNY 10,000)	Sewage discharge of industrial and domestic waste water by region (10 000 tons): Undesired output
Water consumption: Water use (100 million cu. m)	

**Table 2:** WUE difference in two study periods (2006–2011, 2012–2020).

Year	WUE
2006	0.6100
2007	0.5981
2008	0.5902
2009	0.5597
2010 2011	0.5822 0.5321
**Avg. 2006–2011**	**0.5787**
2012	0.5379
2013	0.5373
2014	0.5292
2015	0.538
2016	0.5186
2017	0.5155
2018	0.4192
2019	0.3647
2020	0.3885
**Avg. 2012–2020**	**0.4832**
**Avg. 2006–2020**	**0.5214**

**Table 3:** Group water efficiency, Meta water efficiency, and TGR in 31 provinces and cities in China.

Province	GWE		MWE		TGR	
	Mean	S.D	Mean	S.D	Mean	S.D
Anhui	0.908	0.1744	0.377	0.0411	0.435	0.1228
Beijing	1.083	0.2551	1.083	0.2551	1	0
Fujian	0.44	0.046	0.44	0.046	1	0
Gansu	0.911	0.1604	0.41	0.0723	0.463	0.1091
Guangdong	0.904	0.1825	0.837	0.1985	0.933	0.1436
Guangxi	0.742	0.2268	0.356	0.0437	0.518	0.1473
Guizhou	0.718	0.201	0.337	0.0676	0.495	0.1258
Hainan	0.358	0.0395	0.358	0.0395	1	0
Hebei	0.619	0.176	0.616	0.1673	0.998	0.0097
Henan	0.649	0.0284	0.322	0.0513	0.497	0.0795
Heilongjiang	0.962	0.2499	0.5	0.2509	0.503	0.1528
Hubei	0.966	0.1642	0.399	0.0418	0.428	0.1006
Hunan	1.151	0.107	0.446	0.0447	0.393	0.0651
Jilin	0.884	0.2746	0.329	0.0784	0.39	0.0855
Jiangsu	0.7	0.1022	0.7	0.1022	1	0
Jiangxi	0.793	0.2032	0.346	0.0364	0.462	0.1259
Liaoning	0.432	0.0928	0.432	0.0928	1	0
Inner Mongolia	1.184	0.0776	0.644	0.2787	0.534	0.2078
Ningxia	0.413	0.0324	0.223	0.0375	0.542	0.0901
Qinghai	0.389	0.0659	0.216	0.0612	0.55	0.1139
Shandong	0.736	0.2141	0.735	0.2135	1	0.0012
Shanxi	0.721	0.1662	0.348	0.0702	0.49	0.0829
Shaanxi	1.49	0.045	1.02	0.1311	0.685	0.087
Shanghai	1.238	0.1853	1.236	0.1854	0.999	0.0055
Sichuan	1.015	0.0688	0.397	0.0401	0.393	0.0486
Tianjin	1.025	0.3383	1.012	0.331	0.99	0.0111
Tibet	0.944	0.2812	0.43	0.3236	0.509	0.349
Xinjiang	0.651	0.2091	0.302	0.064	0.483	0.1037
Yunnan	0.601	0.2029	0.346	0.0916	0.591	0.0935
Zhejiang	0.558	0.082	0.558	0.082	1	0
Chongqing	0.973	0.1878	0.408	0.0766	0.426	0.0638

**Table 4:** Average MLI, EC, and TC in three different regions of China (2006–2020).

Region	Province	MLI	EC	TC.
Central	Anhui	1.1097	0.9899	1.125
Central	Henan	1.079	0.9656	1.1212
Central	Heilongjiang	1.0768	0.9096	1.1938
Central	Hubei	1.1235	0.9886	1.1396
Central	Hunan	1.1153	0.9843	1.1404
Central	Jilin	1.1096	0.9758	1.1324
Central	Jiangxi	1.0792	0.9895	1.0951
Central	Shanxi	1.0944	0.9759	1.1255
**Ave. Central**		**1.0984**	**0.9724**	**1.1341**
East	Beijing	1.1214	1.0573	1.1527
East	Fujian	1.1317	0.996	1.1438
East	Guangdong	1.0996	0.9548	1.1554
East	Hainan	1.1045	0.9825	1.1364
East	Hebei	1.1633	0.9879	1.1812
East	Jiangsu	1.1927	1.0012	1.2098
East	Liaoning	1.0793	0.9753	1.116
East	Shandong	1.111	0.937	1.2029
East	Shanghai	1.1063	1.0223	1.084
East	Tianjin	1.0715	0.9409	1.1734
East	Zhejiang	1.1313	0.9724	1.1689
**Ave. East**		**1.1193**	**0.9843**	**1.1568**
West	Gansu	1.1218	0.9689	1.1683
West	Guangxi	1.1167	0.9909	1.1255
West	Guizhou	1.1501	0.9891	1.1654
West	Inner Mongolia	1.1725	0.921	1.2896
West	Ningxia	1.0947	0.9934	1.094
West	Qinghai	1.0782	0.9935	1.091
West	Shaanxi	1.2182	1.0206	1.2287
West	Sichuan	1.1155	0.9927	1.1302
West	Tibet	1.359	0.9064	1.5836
West	Xinjiang	1.0806	0.962	1.1245
West	Yunnan	1.1464	0.9787	1.1823
West	Chongqing	1.1439	1.0182	1.1384
**Ave. West**		**1.1498**	**0.9779**	**1.1935**

**Table 5:** The Mann–Whitney U table to indicate significant statistical differences between the WUE and productivity results for two time periods (2006–2011 and 2012–2020).

Hypothesis Test Summary				
	Null Hypothesis	Test	Sig.	Decision
1	The distribution of Avg. WUE is identical for both time periods.	Independent-Samples Mann–Whitney U Test	0.003 ^a^	Reject the null hypothesis.
2	The distribution of Avg. MLI is identical for both time periods.	Independent-Samples Mann–Whitney U Test	0.066 ^a^	Retain the null hypothesis.
3	The distribution of Avg. TC is identical for both time periods.	Independent-Samples Mann–Whitney U Test	0.234 ^a^	Retain the null hypothesis
4	The distribution of Avg. EC is identical for both time periods.	Independent-Samples Mann–Whitney U Test	0.278 ^a^	Retain the null hypothesis

^a^ Asymptotic significances are displayed. The significance level is 0.050.

**Table 6:** The Kruskal–Wallis test indicates the significant statistical difference between WUE and productivity results for the three regions of China.

Hypothesis Test Summary				
	Null Hypothesis	Test	Sig.	Decision
1	The distribution of Avg. WUE is identical across China’s three distinct regions.	Independent-Samples Kruskal–Wallis Test	0.002	Reject the null hypothesis.
2	The distribution of Avg. MI change is identical across China’s three distinct regions.	Independent-Samples Kruskal–Wallis Test	0.000	Reject the null hypothesis
3	The distribution of Avg. Technology is identical across China’s three distinct regions.	Independent-Samples Kruskal–Wallis Test	0.007	Reject the null hypothesis
4	The distribution of Avg. Efficiency change is identical across China’s three distinct regions.	Independent-Samples Kruskal–Wallis Test	0.350	Retain the null hypothesis

Asymptotic significances are displayed. The significance level is 0.050.

## Data Availability

The data for this research were retrieved from the China Statistical Year Book. The data are openly available at http://www.stats.gov.cn/tjsj/ndsj/, accessed on 7 September 2022.

## Appendix A

**Table A1:** Regional distribution in China.

East	Central	West
Beijing	Anhui	Gansu
Fujian	Henan	Guangxi
Guangdong	Heilongjiang	Guizhou
Hainan	Hubei	Inner Mongolia
Hebei	Hunan	Ningxia
Jiangsu	Jilin	Qinghai
Liaoning	Jiangxi	Shaanxi
Shandong	Shanxi	Sichuan
Shanghai		Tibet
Tianjin		Xinjiang
Zhejiang		Yunnan
		Chongqing

**Table A2:** Meta frontier of all three regions over the period 2006–2020.

Year	East	Central	West
2006	0.7959	0.4784	0.5273
2007	0.8019	0.4668	0.4988
2008	0.7876	0.4159	0.5256
2009	0.7857	0.3894	0.4661
2010	0.8414	0.4499	0.4328
2011	0.7511	0.3989	0.4203
2012	0.7424	0.3949	0.4457
2013	0.7475	0.3949	0.4395
2014	0.7341	0.3841	0.438
2015	0.8017	0.3698	0.4085
2016	0.7286	0.3906	0.4113
2017	0.7342	0.3899	0.3987
2018	0.6167	0.2893	0.3247
2019	0.4999	0.2626	0.3088
2020	0.5504	0.274	0.3164
**Avg.**	**0.7279**	**0.3833**	**0.4242**

**Table A3:** Group frontier of all three regions over the period 2006–2020.

Year	East	Central	West
2006	0.7968	0.7183	0.8995
2007	0.8022	0.7976	0.9073
2008	0.7876	0.8146	0.88
2009	0.7879	0.9444	0.852
2010	0.8461	0.9317	0.8688
2011	0.7557	0.9839	0.8015
2012	0.7453	0.985	0.7634
2013	0.7502	0.9618	0.7978
2014	0.7367	0.9535	0.7799
2015	0.8042	0.9399	0.7763
2016	0.7299	0.9267	0.7745
2017	0.7543	0.8924	0.8454
2018	0.6488	0.7938	0.8609
2019	0.5392	0.768	0.8788
2020	0.5504	0.7735	0.8552
**Avg.**	**0.7357**	**0.879**	**0.8361**
